# Comorbidities’ potential impacts on severe and non-severe patients with COVID-19: A systematic review and meta-analysis

**DOI:** 10.1097/MD.0000000000024971

**Published:** 2021-03-26

**Authors:** Sixiang Cheng, Yuxin Zhao, Fenxiao Wang, Yan Chen, Atipatsa Chiwanda Kaminga, Huilan Xu

**Affiliations:** a Department of Social Medicine and Health Management, Xiangya School of Public Health, Central South University, Changsha, Hunan Province; b College of Data Science and Information Engineering, Guizhou Minzu University, Guiyang, Guizhou Province; c Department of Epidemiology and Health Statistics, Xiangya School of Public Health, Central South University, Changsha, Hunan, China.; d Department of Mathematics and Statistics, Mzuzu University, Malawi, Luwinga, Africa.

**Keywords:** comorbidities, COVID-19, epidemiological, meta-analysis, SARS-CoV-2

## Abstract

**Background:**

An ongoing outbreak of pneumonia associated with the severe acute respiratory coronavirus (SARS-CoV-2) emerged in December 2019 in Wuhan, China. Epidemiologic evidence suggests that patients with comorbidities and novel coronavirus disease 2019 (COVID-19) infection may have poor survival outcomes. However, the risk of these coexisting medical conditions in severe and non-severe cases has not been systematically reported.

**Purpose:**

The present study aimed to estimate the association of chronic comorbidities in severe and non-severe cases.

**Methods:**

A literature search was conducted using the databases PubMed, Embase, China National Knowledge Infrastructure (CNKI), and Wanfang Database, Chinese Scientific Journals Full-text Database (CQVIP) from the inception dates to April 1, 2020, to identify cohort studies assessing comorbidity and risk of adverse outcome. Either a fixed- or random-effects model was used to calculate the overall combined risk estimates.

**Results:**

A total of 22 studies involving 3286 patients with laboratory-confirmed COVID-19 were included in the analysis. Overall, compared with the patients with non-severe cases, the pooled odds ratios (ORs) of hypertension, diabetes mellitus, and cardiovascular, cerebrovascular, and respiratory diseases in patients with severe cases were 2.79 (95% confidence intervals [95% CI]: 1.66–4.69), 1.64 (95% CI: 2.30–1.08), 1.79 (95% CI: 1.08–2.96), 3.92 (95% CI: 2.45–6.28), and 1.98 (95% CI: 1.26–3.12), respectively.

**Conclusions:**

This meta-analysis supports the finding that chronic comorbidities may contribute to severe outcome in patients with COVID-19. According to the findings of the present study, old age and 2 or more comorbidities are significantly impactful to COVID-19 outcomes in hospitalized patients in China.

## Abstract 2

Supplemental Digital Content is available in the text

## 1 Introduction

In December 2019, a series of pneumonia cases with an unknown cause emerged in Wuhan, Hubei province, China, with clinical presentations greatly resembling viral pneumonia.^[1]^ A novel coronavirus was identified as the causative agent, and subsequently termed novel coronavirus disease 2019 (COVID-19) by the World Health Organization (WHO) in February. Since December 2019, the rapid outbreak of COVID-19, which arose from severe acute respiratory syndrome coronavirus (SARS-CoV-2) infection, has recently become a public health emergency of global concern.^[2]^ The pathogen has been identified as a novel enveloped RNA beta coronavirus that has currently been named SARS-CoV-2, which has a phylogenetic similarity to SARS-CoV.^[3]^ Considering its highly epidemical nature, COVID-19 has been initially categorized as Class B infectious disease as stipulated in the Law of the People's Republic of China on Prevention and Treatment of Infectious Diseases and is managed as Class A infectious disease.^[4]^ In the past 2 months, the Chinese government and researchers have conducted extraordinary public health measures to control the epidemic.^[5]^ The WHO declared COVID-19 a Public Health Emergency of International Concern as of February 1, 2020. In the past 2 months, COVID-19 has developed into a worldwide pandemic, with 413,467 confirmed documented cases and 18,433 deaths globally as of March 24, 2020.^[6]^

Epidemiologic evidence suggests that patients with COVID at risk of severe disease outcomes are elderly subjects and those with certain underlying diseases. The presence of these comorbidities may predispose patients with COVID-19 to severe complications of viral infection. During early transmission, on January 24, Huang et al first reported^[7]^ that 41 patients admitted to the designated hospital (Jinyintan) in Wuhan were confirmed to have COVID-19 on January 2, 2020. Of the 41 patients in this cohort, 22 (55%) developed severe dyspnea, 13 (32%) required admission to an intensive care unit (ICU), and 6 died. Of the 41 confirmed patients, 13 (32%) had underlying diseases, including cardiovascular disease, diabetes, hypertension, and chronic obstructive pulmonary disease and malignancy. Subsequently, on January 30, Chen et al^[8]^ reported the epidemiological and clinical features of 99 patients treated at designated hospitals and found that 55.5% of patients with chronic underlying diseases were more susceptible to SARS-CoV-2. On February 7, Wang et al analyzed^[9]^ 138 hospitalized patients with COVID-19 and found that 64 (46.4%) patients have underlying comorbidities. Most importantly, compared with patients who did not require ICU admission, patients who required ICU admission were more likely to have underlying comorbidities. It may be worth noting that patients with severe cases admitted to the ICU had a significantly higher proportion of comorbidities (72.2%) than those not admitted to the ICU (37.3%). This solid evidence suggests that complications in patients with COVID-19 may be risk factors for patients’ poor clinical outcomes (severe/critical condition, critical care, or death).^[10]^ To acquire more accurate conclusions on the clinical characteristics and obtain more convincing evidence, a systematic evaluation and detailed estimate for the overall comorbidities in severe and non-severe COVID-19 cases may aid the public health sector in developing policies for surveillance, preparedness, and response to the infection and its severe outcomes.

The present study was conducted to evaluate the association of coexisting chronic conditions in patients with non-severe and severe cases. Thus, a systematic review and meta-analysis of published limited literature were conducted to explore their possible contributions to the severity and complication of COVID-19.

## 2 Materials and methods

### 2.1 Ethics committee approval

This project did not require institutional review board approval because it used publicly accessible information and data of published studies, which already obtained consent from participants and have been approved by other ethics review boards.

### 2.2 Search strategy and selection criteria

This systematic review and meta-analysis have been reported in accordance with the Preferred Reporting Items for Systematic Reviews and Meta-analysis guidelines.^[11]^ PubMed, Embase, China National Knowledge Infrastructure, Wanfang Database, Chinese Scientific Journals Full-text Database (CQVIP) were searched from the inception dates to April 1, 2020, to identify cohort studies. The following search terms (MeSH) were used: “2019 novel coronavirus, novel coronavirus-infected pneumonia (NCIP), COVID-19,” “2019-nCoV” AND “comorbidities,” “clinical characteristics,” “clinical findings,” “clinical features,” “clinical “outcome,” and “epidemiological” without any language restriction. If multiple languages were used to describe and publish the same data, the English version was selected. The data from all included studies were extracted and reviewed independently by XFW and SXC. Any dispute or disagreement between the 2 reviewers that could not be agreed upon was arbitrated by a third reviewer (HLX). The detailed search strategy can be found in Table S1 (see Table S1 in Supplemental Content, http://links.lww.com/MD/F821, which demonstrates the search strategy of included studies).

The inclusion criteria were as follows:

(1) studies describing the epidemiological and clinical features of COVID-19;
(2) studies providing data of patients with severe and non-severe cases; and
(3) studies written in English or Chinese.

The following studies were excluded:

(1) case reports, reviews, editorials, expert opinions, letters, conference abstracts, and animal trials and family-based studies;
(2) studies without usable or sufficient data; and
(3) studies on children and pregnant women.

### 2.3 Definitions

The types of COVID-19 severity was determined according to the Guidelines Novel coronavirus pneumonia diagnosis and treatment (version 5.0).^[12]^ Briefly, the criteria for disease severity is classified as follows:

(1) Mild cases: The clinical symptoms are mild, with no abnormal radiological findings.
(2) Moderate cases: Fever, cough, and other symptoms are present with pneumonia on chest CT.
(3) Severe cases: The disease is classified as severe if one of the following conditions is met: respiratory distress, respiratory rate > 30/minute. Oxygen saturation on room air at rest <93%. The partial pressure of oxygen in arterial blood/FiO_2_ < 300 mm Hg.
(4) Critical cases: One of the following conditions has to be met: Respiratory failure occurs and mechanical ventilation is required. Another organ dysfunction is present, requiring ICU monitoring and treatment. Most patients received antibacterial therapy, including moxifloxacin, ceftriaxone, and azithromycin. More patients received antiviral agents within the first 4 days in the moderate group than in the severe group, patients received antibiotics and corticosteroids in the critical and severe groups.

### 2.4 Data extraction

Data extracted from selected studies included the first author's name; publication date; regions; dates of the recruitment and follow-up; total sample size (divided by sex); age estimates (from the reported mean, median, or midpoint for the age range of the highest subject frequency); number of participants with severe and non-severe cases; and number of comorbidities including hypertension, diabetes (both types I and II, if separately mentioned), cardiovascular disease, respiratory disease, and cerebrovascular disease. If necessary, attempts to contact the research author for additional missing information were also made.

### 2.5 Quality assessment

The methodological quality of all included studies was independently assessed using the modified version of the Newcastle-Ottawa Scale (NOS). The study quality would also be evaluated based on the NOS score. Nine items had been determined in all studies, and each of them had been assigned a score of 1. Specifically, the total scores of each study were between 0 and 9, and a study with a score ≥7 would be deemed to have high quality.

### 2.6 Statistical analysis

Microsoft Office 2010 and Excel 2010 software were used to organize the incorporated literature and data, and all statistical analyses in this study were performed using Stata 12.0 (StataCorp LP, College Station, TX). The odds ratio (OR) (95% confidence intervals [95% CI]) was used to summarize the effect sizes of ORs to describe the ratio of the probability of COVID-19 in patients with severe and non-severe cases. The heterogeneity between studies was quantified by the *I*^2^ statistic and evaluated by the *Q* test. An *I*^2^ > 50% and *P* < .05 indicated the existence of significant heterogeneity. Therefore, a random-effects model was used to summarize the effect sizes (ORs),^[13]^ when significant heterogeneity was observed; otherwise, a fixed-effects model was used. The potential publication bias was assessed by Begg's funnel plot and Begg's test. Subgroup analysis was conducted to explore the potential heterogeneity between studies with different subject characteristics.

## 3 Results

### 3.1 Study identification and selection

**Figure 1:**
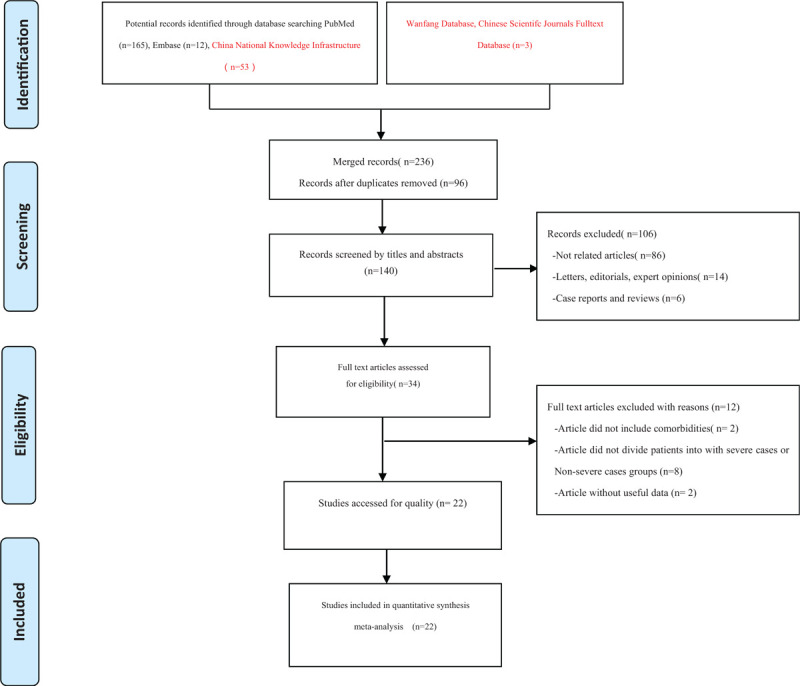
Flow diagram of included/excluded studies. A total of 236 documents were found in the initial search. After removing duplicates, reading titles, abstracts and full texts, and evaluating the quality of the articles, 22 eligible studies were included and analyzed.

A total of 236 articles were identified through a preliminary database search. After the initial screening of abstracts and titles, 96 duplicates were excluded using EndNote X8 (Thomson Reuters, MI). An assessment of 140 articles identified 34 articles for further review. After reading these documents carefully, 12 articles were excluded (8 articles did not divide patients into the non-severe and severe groups, 2 articles involved elderly patients, and 2 articles did not supply applicable data). Finally, as of March 25, 2020, 22 eligible studies involving a total of 3286 patients met the inclusion criteria in this meta-analysis. The detailed screening process is shown in Figure 1.

### 3.2 Study characteristics

Systematic analysis of studies that described the epidemiological, demographic, and clinical features of patients with COVID-19 and reported the number of chronic diseases has identified 22 reports^[7–10,14–32]^ with 3286 patients. The majority confirmed the cases were from hospitals of Hubei province, particularly in Wuhan. All studies were published between January 14 and March 30, 2020. Of these, 19 were of moderate and 3 were of high quality. The baseline information about the included studies is presented in Table 1.

**Table 1:** Characteristics of 22 included studies.

				Gender					Comorbidities (all n)						
First author	Dates (mm. yy)	Region	Patients	M	F	Age (yr)	Non-severe patients (n)	Severe patients (n)	Hypertension	Diabetes	Cardiovascular disease	Respiratory system diseases	Cerebrovascular disease	Study design	Quality score
Huang et al^[7]^	2019.12.16–1.2	Wuhan	41	30	11	49	13	28	6	8	6	2		Retrospective cohort study	8
Guan et al^[8]^	2019.12.11–1.29	Wuhan	1099	640	459	34–57	926	173	165	81	27	12	15	Retrospective cohort study	8
Wang et al^[9]^	01.1–2.3	Wuhan	138	75	63	45.7	36	102	43	14	20	4	7	Retrospective cohort study	7
Wang et al^[13]^	1.29–2.16	Wuhan	69	32	37	35.0–62.0	14	55	9	7	8			Retrospective cohort study	6
Zhang et al^[14]^	01.06–02.03	Wuhan	140	71	69	57.0	82	58	42	17	7	2	4	Retrospective cohort study	7
Zhao et al^[15]^	1.23–2.5	Wuhan	34	17	17	27–56	19	15	3					Retrospective cohort study	6
Zhou et al^[16]^	12.29–1.31	Wuhan	119	72	119	46.0–67	54	137	58	36	15	6		Retrospective cohort study	7
Cheng et al^[17]^	1.23–2.6	Wuhan	463	244	219	43 ± 60	282	181	107	40	28	19	14	Retrospective cohort study	7
Xiong et al^[18]^	1.17–2.20	Wuhan	90	41	49	53.0 ± 16.9	58	31	26	14	6	5	6	Retrospective cohort study	7
Yang et al^[19]^	1.24–2.11	Wuhan	52	20	32	59·7 ± 13.3	20	32	9	5	4	7		Retrospective cohort study	6
Wu et al^[20]^	1.22–2.14	Jiangsu	80	41	39	46.10 ± 15.42	28	52	5	25	1			Retrospective cohort study	6
Xu et al^[21]^	12.30–01.26	Zhejiang	62	35	27	37 ± 54	33	29	5	1		1	1	Retrospective cohort study	6
Wang et al^[22]^	1.11–2.29	Shenzhen	55	22		2–69	53	2	8					Retrospective cohort study	6
Suo et al^[23]^	1.31–2.10	Wuhan	50	21	25	35 ± 62	21	25	24	16	12		11	Retrospective cohort study	
Zhang et al^[24]^	1.21–2.11	Beijing	74	35	39	52.7 ± 19.1	56	18	13	9	3	5	2	Retrospective cohort study	6
Fang et al^[25]^	1.21–2.18	Anhui	79	34	45	45.1 ± 16.6	55	24	16	8	3		3	Retrospective cohort study	6
Yuan et al^[26]^	1.24–2.23	Chongqing	223	105	NA	46.5 ± 16.1	192	31	25	18	2	1		Retrospective cohort study	7
Li et al^[27]^	1.20–2.27	Zhuzhou	40	40	40	47.8 ± 19.5	17	63	14					Retrospective cohort study	6
Gong et al^[28]^	1.23–2.09	Chongqing	80	45	45	54.20 ± 12.7	65	15	8	6				Retrospective cohort study	6
Xiang et al^[29]^	1.21–1.27	Jiangxi	49	33	16	42.9	40	9	2	4				Retrospective cohort study	6
An et al^[42]^	12.25–1.26	Wuhan	201	128	NA	43–60	117	84	23	16				Retrospective cohort study	7
Li et al^[31]^	1.20–2.10	Guangzhou	66	29	37	58	13	31	10	5	1			Retrospective cohort study	6

### 3.3 Association between hypertension and severe cases between non-severe cases

A total of 3286 patients from 22 studies were included to detect the relationship between hypertension and adverse outcome. The random-effects model was used due to significant heterogeneity among the preceding studies (*I*^2^ = 83.5%, *P* = .000). The results showed that patients with hypertension were more likely to have poor outcomes than patients without hypertension (pooled OR = 2.79; 95% CI: 1.66–4.69) (Fig. 2    A).

**Figure 2:**
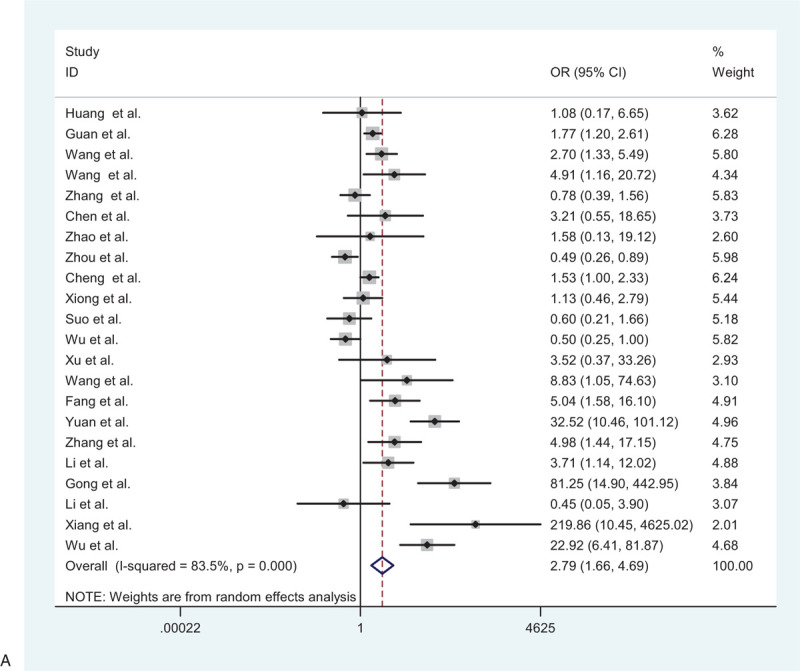
(A) Forest plot for the association between hypertension and severe patients and non-severe patients. (B) Forest plot for the association between diabetes and severe patients and non-severe patients. (C) Forest plot for the association between cardiovascular disease and severe patients and non-severe patients. (D) Forest plot for the association between cerebrovascular disease and severe patients and non-severe patients. (E) Forest plot for the association between respiratory system disease and severe patients and non-severe patients.

**Figure 2 (Continued):**
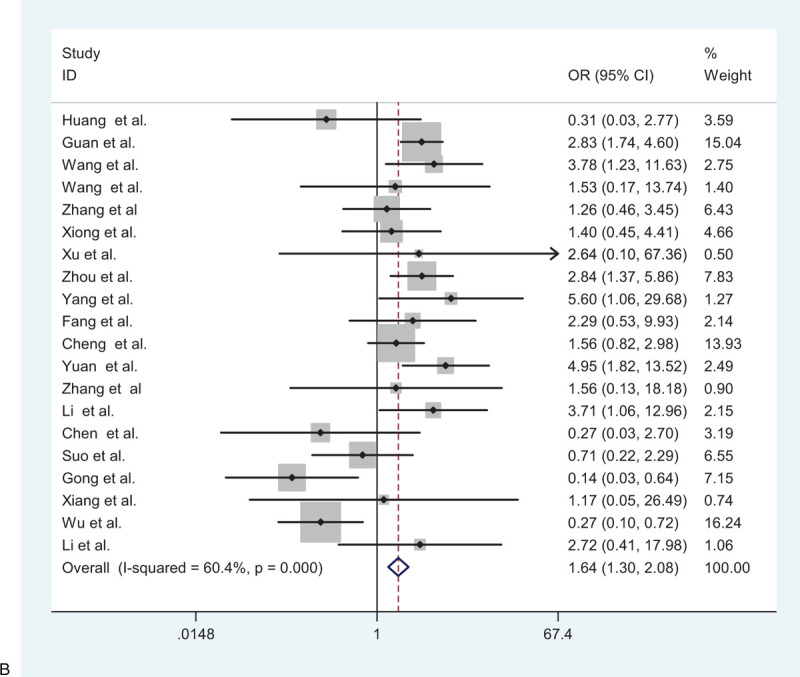
(A) Forest plot for the association between hypertension and severe patients and non-severe patients. (B) Forest plot for the association between diabetes and severe patients and non-severe patients. (C) Forest plot for the association between cardiovascular disease and severe patients and non-severe patients. (D) Forest plot for the association between cerebrovascular disease and severe patients and non-severe patients. (E) Forest plot for the association between respiratory system disease and severe patients and non-severe patients.

**Figure 2 (Continued):**
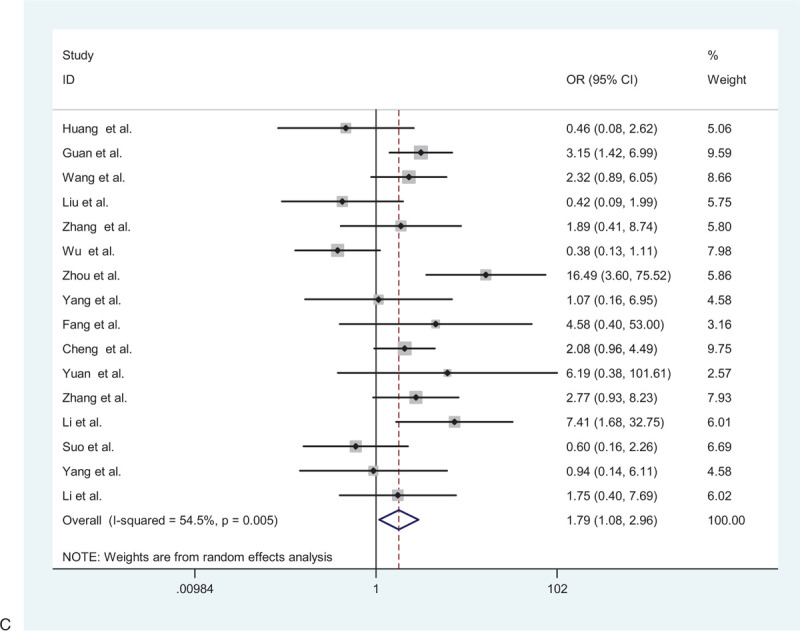
(A) Forest plot for the association between hypertension and severe patients and non-severe patients. (B) Forest plot for the association between diabetes and severe patients and non-severe patients. (C) Forest plot for the association between cardiovascular disease and severe patients and non-severe patients. (D) Forest plot for the association between cerebrovascular disease and severe patients and non-severe patients. (E) Forest plot for the association between respiratory system disease and severe patients and non-severe patients.

**Figure 2 (Continued):**
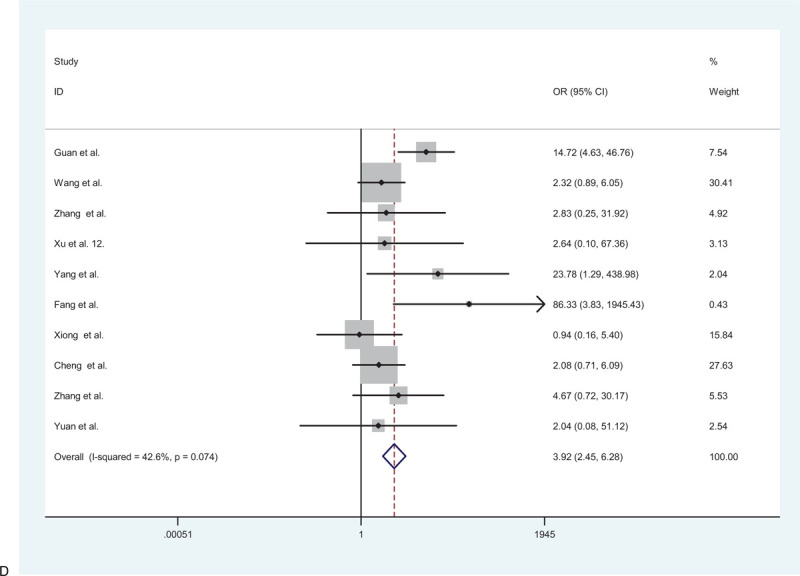
(A) Forest plot for the association between hypertension and severe patients and non-severe patients. (B) Forest plot for the association between diabetes and severe patients and non-severe patients. (C) Forest plot for the association between cardiovascular disease and severe patients and non-severe patients. (D) Forest plot for the association between cerebrovascular disease and severe patients and non-severe patients. (E) Forest plot for the association between respiratory system disease and severe patients and non-severe patients.

**Figure 2 (Continued):**
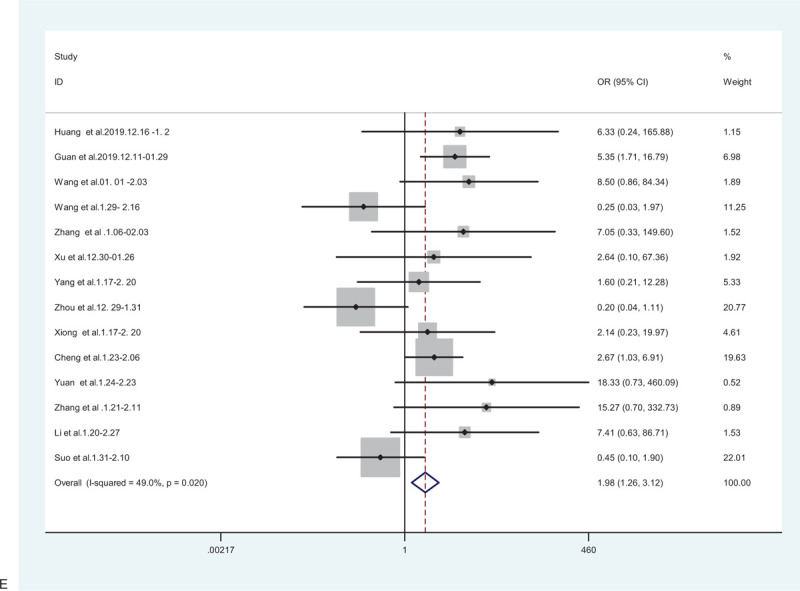
(A) Forest plot for the association between hypertension and severe patients and non-severe patients. (B) Forest plot for the association between diabetes and severe patients and non-severe patients. (C) Forest plot for the association between cardiovascular disease and severe patients and non-severe patients. (D) Forest plot for the association between cerebrovascular disease and severe patients and non-severe patients. (E) Forest plot for the association between respiratory system disease and severe patients and non-severe patients.

### 3.4 Association between diabetes mellitus and severe/non-severe cases

Among the studies included in the meta-analysis, 20 studies, comprising 3271 patients, evaluated the correlation between diabetes and adverse outcome. A random-effects model was used to estimate the correlation because significant heterogeneity among patients with diabetes mellitus was more susceptible to the development of critical illness than patients without diabetes mellitus (pooled OR = 1.64; 95% CI: 1.30–2.08) (Fig. 2    B).

### 3.5 Association between cardiovascular diseases and severe/non-severe cases

A total of 2877 patients from 16 studies were included to determine the relationship between cardiovascular diseases and adverse outcomes. Therefore, the random-effects model was used due to significant heterogeneity among the preceding studies (*I*^2^ = 54.5%, *P* = .005). The results showed that patients with cardiovascular disease were more likely to have poor outcomes than those without cardiovascular disease (pooled OR = 1.79; 95% CI: 1.08–2.96) (Fig. 2    C).

### 3.6 Association between cerebrovascular disease and severe/non-severe cases

A total of 2399 patients from 10 studies were included to determine the relationship between cerebrovascular disease and adverse outcomes. Therefore, the random-effects model was used due to significant heterogeneity among the preceding studies (*I*^2^ = 42.5%, *P* = .074). The results showed that patients with the cerebrovascular disease were more likely to have poor outcomes than those without the cerebrovascular disease (pooled OR = 3.92; 95% CI: 2.45–6.28) (Fig. 2    D).

### 3.7 Association between respiratory disease and severe/non-severe cases

Positive associations were also observed in 14 studies with 2767 patients, a random-effects model was used to estimate the correlation because significant heterogeneity among these studies existed (*I*^2^ = 49.0%, *P* = .020). Therefore, the results revealed that patients with respiratory disease were more likely to have poor outcomes than those without the respiratory disease (pooled OR = 1.98; 95% CI: 1.26–3.12) (Fig. 2    E).

### 3.8 Subgroup and sensitivity analyses

In the outcome and subgroup analyses after combining the ORs reported in the 22 included studies, it was found that complications were associated with the risk of disease severity in all patients with COVID-19 in the random-effects model. The pooled OR was 2.45 (95% CI: 1.33–4.52), and substantial heterogeneity was detected (*I*^2^ = 88.3%, *P* < .001) (Fig. 2    A). Subgroup analyses were conducted to identify potential heterogeneity moderators for the association between underlying diseases and the risk of adverse outcomes. After subgroup analyses, it was found that studies in non-Wuhan regions (OR = 8.11; 95% CI: 5.54–11.88) and sample size ≤ 100 (OR = 2.34; 95% CI: 1.88–2.92) were identified as the 2 most relevant heterogeneity moderators for the association between comorbidities and disease severity in patients with COVID-19. This information is summarized in Table 2.

**Table 2:** Subgroup analysis of heterogeneity different study characteristics.

				Heterogeneity		Egger test		
Study	No. of studies	No. of patients	Pooled OR (95% CI)	*I*^2^ (%)	*P* value	*t*	*P*	Std. Err.
Study region								
Wuhan	12	2541	1.21 (0.99, 1.48)	66.7	.001	2.48	.022	0.821
Non-Wuhan	10	811	8.11 (5.54, 11.88)	73.1	.000			
Age of subjects	16	2723	1.34 (1.15, 1.57)	70.4	.000			
Mean < 50				73.4	.003	2.33	.032	0.722
Mean > 50	6	107	1.63 (1.19, 2.23)					
Sample size								
Number ≤ 100	7	2455	1.33 (1.13, 1.56)	89.3	.001	2.16	.044	0.700
Number ≤ 100	15	831	2.34 (1.88, 2.92)	52.7	.000			
NOS score	8	1149						
NOS = 6			1.46 (1.19, 1.77)	84.5%	.000	2.16	.044	0.700
NOS = 8	3	1278	1.71 (1.31, 2.23)	0	.605			
NOS = 7	11	779	1.75 (1.42, 2.18)	84.8	.605			

95% CI = 95% confidence intervals, NOS = Newcastle-Ottawa scale, OR = odds ratio.

**Figure 3:**
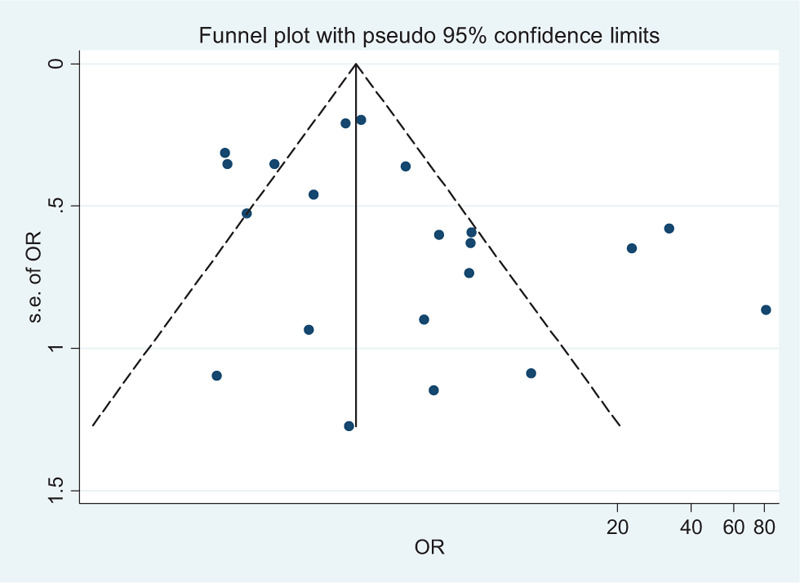
Funnel plots of the 22 studies included in the meta-analysis.

Sensitivity analysis was performed after excluding 3 studies, the pooled OR was 1.64 (1.44–1.88), which was slightly but not significantly lower than the previous (pooled OR 1.62; 95% CI: 1.42–1.84), indicating the results of our studies were reliable (Fig. 3).

### 3.9 Publication bias

**Figure 4:**
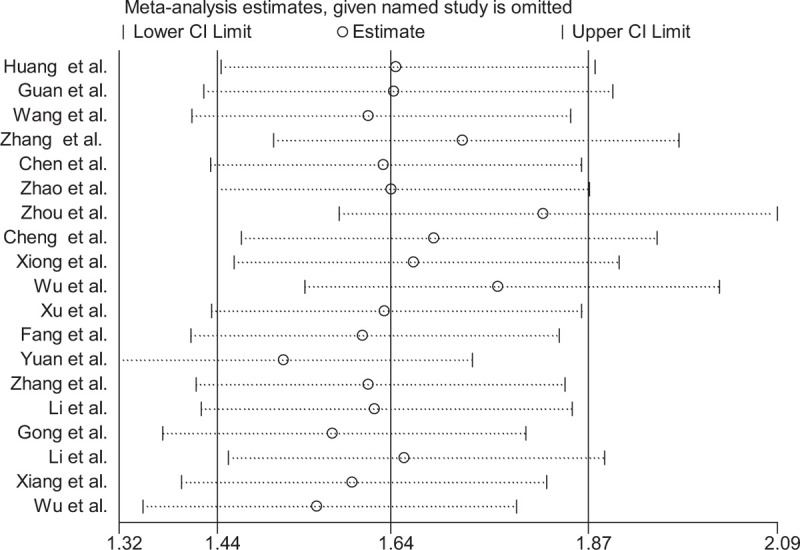
Sensitivity analysis of comorbidities and risk in patients of COVID-19 between severe patients and non-severe patients.

The results of Egger's test are revealed in terms of the potential risk of publication bias coefficient (*t* = 1.86, *P* = .078). The funnel plot in Figure 4 did not show any substantial asymmetry.

## 4 Discussion

The present study was conducted to systematically evaluate the relationship between underlying diseases and non-severe and severe COVID-19 cases. Guan et al reported that patients with at least 1 comorbidity had poor clinical outcomes.^[31]^ The meta-analysis was based on data from 22 studies with the laboratory-confirmed and clinical diagnosis of COVID-19. All cases were from hospitals in China. Twelve studies were conducted in Wuhan designated hospitals. Regarding the geographical distribution, the number of patients with underlying diseases in Hubei Province is also higher. In these studies, a higher number of men than women with COVID-19 were observed. The possible explanation is that COVID-19 in patients in the previous study was related to exposure associated with the Huanan Seafood Wholesale Market, and most affected patients were male workers.^[9]^ Similarly, MERS-CoV and SARS-CoV have also been found to infect more men than women.^[32,33]^ Guan et al reported that old age and the presence of comorbidity might be associated with increased mortality. In critical patients (16%), nearly a third had underlying illnesses. If they had more than 2 types of underlying diseases, the survival situation will become even worse.^[31]^ When populations with low immune function, such as elderly individuals and patients with diabetes, HIV infection, and long term use of immunosuppressive agents are infected with COVID-19^[32,33]^ prompt administration of antibiotics to prevent infection and strengthening of immune support treatment might reduce complications and mortality. In addition, metabolic disorders may lead to low immune function by impairing macrophage and lymphocyte function, which may make individuals more susceptible to disease complications. Besides, metabolic disorders may lead to low immune function by impairing macrophage and lymphocyte function, which may make patients more susceptible to disease complications.^[32]^ Considering antibiotics prone to develop resistance, the clinical doctors should national use antibiotics and inappropriate use of antibiotics should be avoided, especially the combined use of broad-spectrum antibiotics.^[33]^

The meta-analysis results of the present study suggest an increased risk of developing severe COVID-19 complications in individuals with hypertension, diabetes, cardiovascular disease, and respiratory disease than in those with other or no underlying chronic diseases. Similar observations were also reported in H1N1,^[33]^ MERS-CoV, and influenza.^[37]^ Two studies reported that compared to subjects with no comorbidities, patients with severe pandemic influenza more likely had cardiovascular diseases (2.92; 95% CI: 1.76–4.86) and hypertension (1.49; 95% CI: 1.10–2.01), 6.43 (95% CI: 3.40–12.17), respectively.^[32–35]^ Recently, An et al reported that, compared with the non-death group, the underlying disease was an important risk factor for death.^[36]^ Nearly 50% of patients who died had complications of more than 2 chronic diseases. Among these patients, 63.6% had complications of hypertension.^[37]^ Similar results, including older age (≥65 years), a high number of underlying comorbidities, and more prominent laboratory examination information abnormalities, were associated with severe cases.^[32,34]^ Lu et al reported^[40]^ that 21 (28.8%) patients who died had diabetes and believed that more attention should be paid to the control of blood glucose levels in patients with COVID-19 with complications of diabetes. For instance, patients with diabetes mellitus (DM), obesity, and/or hypertension and COVID-19 have increased mortality and morbidity rates.^[16,17,37,8,38,32,34,39–42,15]^ A number of literature reports have documented cardiovascular disease, hypertension, chronic obstructive pulmonary disease, and severe asthma, diabetes, kidney failure, severe liver disease, immunodeficiency, and malignancy may confer an increased risk of adverse outcomes. In addition, in non-elderly populations, the more prevalent of above co-conditions is cardiovascular disease and hypertension, with a prevalence of approximately 10% in the 20 to 39 age group and 38% in the 40 to 59 age group in the USA and similarly high percentages in many other countries.^[43]^

In April, a meta-analysis of 12 studies from China including 2389 COVID-19 patients (674 severe cases) showed that hypertension patients carried a nearly 3.48-fold higher risk of dying from COVID-19 (95% CI: 1.72–7.08). The severity rate of COVID-19 in hypertensive patients was much higher than in non-hypertensive cases (37.58% vs 19.73%, pooled OR: 2.27; 95% CI: 1.80–2.86). Meanwhile, the pooled ORs of COVID-19 fatality for hypertension vs non-hypertension were 6.43 (95% CI: 3.40–12.17) and 2.66 (95% CI: 1.27–5.57) in age <50 years and ≥50 years patients, respectively.^[44]^ Another analysis also revealed that compared to COVID-19 patients with no preexisting chronic cardiovascular condition, COVID-19 patients who present with either hypertension or CVD have an approximately 3- to 4-fold higher risk of developing severe disease, with an OR of 2.92 (95% CI: 2.35, 3.64) and 3.84 (95% CI: 2.90, 5.07), respectively.^[45]^

Recently, 2 meta-analyses further confirmed the positive association between diabetes and COVID-19 severity (pooled OR = 2.58; 95% CI: 1.93–3.45). Moreover, patients with diabetes infected with SARS-CoV-2 had a 2.95-fold higher risk of fatality compared with those patients without diabetes (95% CI: 1.93–4.53).^[46]^ The results also showed patients with diabetes with SARS-CoV-2 infection had a higher risk of developing severe COVID-19 (pooled OR = 2.58; 95% CI: 1.93–3.45), patients with diabetes were 2.73 (95% CI: 1.95–3.82), 2.98 (95% CI: 1.49–5.98), and 2.08 (95% CI: 1.38–3.15) times more likely to progress to severe manifestations, ICU admission, and death. Having cerebrovascular disease also significantly increased the risk of severe manifestations, ICU admission, and death, with ORs of 2.24 (95% CI: 1.26–3.98), 20.20 (95% CI: 2.34–174.44), and 13.27 (95% CI: 0.71–249.04), respectively.^[47]^ Our research also achieved similar results. Of note, these findings have provided further objective evidence.

As the coronavirus disease, 2019 (COVID-19) pandemic has spread widely around the globe,^[48]^ the knowledge of COVID-19 mortality rates for people <65 years old and non-elderly individuals without underlying diseases in pandemic epicenters at the population should be discussed. Recently John and his colleges used cross-sectional data of 13 countries and 14 US states with at least 800 COVID-19 deaths as of April 24, 2020, and with information on the number of deaths in people with age < 65. They found the people <65 years old had 30- to 100-fold lower risk of COVID-19 death than those 65 years old in 11 European countries and Canada, 16- to 52-fold lower risk in US locations, and less than 10-fold in India and Mexico.^[49]^ Their study indicated that people <65 years old have very small risks of COVID-19 death even in pandemic epicenters and deaths for people <65 years without underlying predisposing conditions are remarkably uncommon. In addition, based on existing data to-date.^[50–52]^

There were made some successful treatment regimens in these cohorts studies. In the case series of 99 hospitalized patients with COVID-19 infection from Wuhan, oxygen was given to 76%, noninvasive ventilation in 13%, mechanical ventilation in 4%, extracorporeal membrane oxygenation (ECMO) in 3%, continuous renal replacement therapy (CRRT) in 9%, antibiotics in 71%, antifungals in 15%, glucocorticoids in 19%, and intravenous immunoglobulin therapy in 27%.^[8]^ Antiviral therapy consisting of oseltamivir, ganciclovir, and lopinavir–ritonavir was given to 75% of the patients. The duration of noninvasive ventilation was 4 to 22 days (median 9 days) and mechanical ventilation for 3 to 20 days (median 17 days). In the case series of children discussed earlier, all children recovered with basic treatment and did not need intensive care.^[53]^ In 476 patients from a multi-center study in Wuhan, Shanghai has found that in the moderate and severe groups, patients who received antibiotics or corticosteroids had longer hospital stays than those who did not. In the critical group, patients usually received early antiviral treatment within the first 4 days, and not giving corticosteroids throughout the hospitalization period was associated with good prognosis; in the severe group, patients received high-flow oxygen treatment. In the critical group, patients received extracorporeal membrane oxygenation rescue therapy and were given invasive mechanical ventilation.^[54]^ More evidence is needed before these drugs are recommended. Other drugs proposed for therapy are intravenous immunoglobulin, interferons, chloroquine, and plasma of patients recovered from COVID-19.^[55–58]^

The new outbreak of the respiratory illness caused by coronavirus has been a global public health crisis since December 2019. This study also was to discuss the similarities and differences between pandemic influenza and the COVID-19 virus. COVID-19 and influenza viruses can both cause respiratory illnesses. They are 2 viruses with common clinical characteristics that cause pneumonia. First, influenza and COVID-19 infections of pneumonia are viral infections and have obvious family members of the aggregation. Both viruses could be transmitted through droplets, contact with contaminated hands, and household appliances. For example, a person with influenza or COVID-19 infections would gradually spread to their families, friends, or other people in crowded places.^[32]^

However, the main difference between these viruses is in their pathogenicity.^[59]^ The main symptoms are fever, headache, muscle pain, and general discomfort. There are many cases of fever; most cases can reach body temperatures between 39°C and 40°C as well as chills, and more accompanied by systemic muscle and joint pains, fatigue, loss of appetite, and other systemic symptoms. It is well known that flu cases often have a sore throat, dry cough, but also nasal congestion, runny nose, chest pain, discomfort, and flushed face.^[35]^ In addition, some COVID-19 cases in Wuhan are characterized by vomiting, abdominal pain, diarrhea, and other gastrointestinal symptoms.^[60]^ On the other hand, based on data from the available medical records in Wuhan, COVID-19 cases are characterized by fever (37.3°C–38°C), weakness, dry cough.^[60]^

Thirdly, the treatment plan is different between influenza and COVID-19 infections. For instance, the treatment of COVID-19 cases now mainly includes social isolation, symptomatic support treatment, and close monitoring of the disease status, especially respiratory frequency, finger oxygen saturation. In this regard, suspected cases should be treated in single-room isolation, whereas confirmed cases can be treated in the same ward. Also, critically ill patients should be admitted to the intensive care unit for special treatment as soon as possible. Additionally, there is no licensed COVID-19 vaccine or treatment until now, although the World Health Organization (WHO) indicated that a number of therapeutic drugs are currently undergoing clinical trials in China, and more than 20 vaccines are under development in the world. On the contrary, there are antiviral drugs and vaccines available for influenza.^[61]^

These findings suggest that age and comorbidities may be risk factors for the development of severe cases. These findings also would be relevant in developing public health measures and practices targeting patients with COVID-19 with comorbidities to avert the poor clinic outcome of the infectious disease. Overall, comorbidities related to metabolic syndrome are thought to be etiologically linked to COVID-19 pathogenesis. These diseases are known to downregulate key mediators of host innate immune response to pathogenesis. For example, diabetes, hyperglycemia, and insulinogenic attenuate the synthesis of pro-inflammatory cytokines, such as IFN-γ and interleukins, to functionally impair the innate and humoral immune systems of the host.^[41]^ Furthermore, cytokine overload related to the Th_1_ to Th_2_ shift in severe viral infection, when accompanied by increased cytokine synthesis from metabolic diseases, can be detrimental in synergistically affecting the endothelium and leads to a range. Several limitations should be noted in this meta-analysis. First, all studies included in this meta-analysis are retrospective studies with large heterogeneity; most patients originated from China, and the present study aimed to use the conclusions of this study to predict patients with general domestic characteristics, including other countries and races, which may have contributed to the heterogeneity observed in the meta-analysis. Other sources of heterogeneity may be related to the large range of sample sizes and different study designs. These factors may levy some limitations on the estimated contribution of chronic diseases to severe COVID-19 cases and render our results as a guide to generate more accurate estimates to examine intervention strategies for infection in patients with chronic diseases. Second, the small number of studies selected did not allow us to evaluate the prevalence of chronic comorbidities in each outcome (e.g., chronic liver disease, immune deficiency, and HIV infection) of severe COVID-19 cases. Additionally, limited information was provided in the original articles on the chronic comorbidities in terms of how long patients had the condition(s), the onset of the condition(s) prior to or following COVID-19 manifestation, and whether the patients have received treatment. The epidemiological data of SARS-CoV-2 infection suggested that severe COVID-19 cases were more likely to be elderly patients with underlying comorbidities (such as diabetes mellitus and hypertension) indicating that age was an important risk factor of severity and fatality of COVID-19. Similarly, the China CDC recently reported that patients aged ≥80 years had the highest case fatality rate, 14.8%, among different age groups.^[34]^

In this situation, the world is facing an unknown novel virus; and in the absence of specific drugs, early identification of severe and critically ill patients and timely intervention are essential to reduce the mortality of COVID-19. The global pandemic caused by COVID-19 is just beginning, and the number of patients infected by the virus is increasing at an exponential rate. Combining these health practices with educating the public on the risk of complications risks can be an effective public health measure for the intervention of infection-related complications particularly in the rapid spread in countries where the epidemic is rising at an alarming speed.

Therefore, based on the above-mentioned limitations, more case–control studies with larger sample sizes and high quality are required to study underlying diseases more relevant than non-severe conditions among different countries and ethnicities.

## 5 Conclusions

This meta-analysis supports the finding that chronic comorbidities may contribute to severe outcome in patients with COVID-19. According to the findings of the present study, old age and 2 or more comorbidities are significantly impactful to COVID-19 outcomes in hospitalized patients in China.

## Acknowledgments

We would like to acknowledge the medical staff who work in the frontline for containing COVID-19 in China. We also take the opportunity to sincerely thank the anonymous reviewers for their thoughtful and meaningful comments.

## Author contributions

SXC and HLX developed the idea for and designed the study and had full access to all data in the study and take responsibility for the integrity of the data and accuracy of the data analysis. SXC and YXZ contributed to the writing of the manuscript. ACK contributed to the critical revision of the manuscript. SXC and FXW contributed to the statistical analysis. All authors contributed to data acquisition, data analysis, and data interpretation and reviewed and approved the final version.

**Conceptualization:** Sixiang Cheng, Huilan Xu.

**Data curation:** Yan Chen.

**Methodology:** Sixiang Cheng, Xiaofen Wang, Huilan Xu.

**Software:** Xiaofen Wang.

**Supervision:** Huilan Xu.

**Validation:** Yuxin Zhao.

**Writing – original draft:** Sixiang Cheng, Yuxin Zhao.

**Writing – review & editing:** Sixiang Cheng, Atipatsa Chiwanda Kaminga.
